# Using the Synthetic Control Method to Evaluate the Impact of a Land-Based Gambling Ban on Crime

**DOI:** 10.1007/s10899-024-10281-y

**Published:** 2024-03-01

**Authors:** Jakub Žofčák, Josef Šíma

**Affiliations:** 1 Department of Economics and Management, Faculty of Social and Economic Studies, Jan Evangelista Purkyně University in Ústí Nad Labem, Ústí Nad Labem, Czech Republic; 2 Department of International Business, Metropolitan University Prague, Prague, Czech Republic

**Keywords:** Gambling, Criminality, Synthetic control method, Panel data, Czech Republic

## Abstract

We use the synthetic control method to examine whether land-based gambling ban has an effect on crime in a given city. In a sample of four Czech cities where the ban was introduced, we show that these cities had roughly the same crime rate development in the years after the ban as the control cities without the ban, showing that there is no significant impact. As this is, to our knowledge, the first use of the synthetic control method in the context of gambling, the main contribution of this study lies in demonstrating the possibilities in its use. Employing this versatile method may improve the current situation where many gambling impact studies suffer from the absence of a control group or an identification strategy to confirm causal relationship. Last but not least, the results of this study make an important contribution to the debate on blanket measures in gambling regulation; although pathological gambling is linked to crime, banning this activity may not lead to the expected consequences.

## Introduction

The relationship between problem gambling and various criminal activities has been confirmed by many studies (see Grinols, 2017; Clark & Walker, 2009; Walker et al., 2010; Lloyd et al., 2014; Perrone et al., 2013, and others) including systematic reviews (Adolphe et al., 2018; Banks, 2017). Regarding the nature of such criminal activities, the most common are non-violent, financial and drug offences, including (but not limited to) fraud, theft, embezzlement and others (see Monash University Centre for Criminology and Criminal Justice 2000; Williams et al., 2005; Abbott & McKenna, 2005; Turner et al., 2009). Violent crimes, such as robberies or burglaries are also mentioned but not so often (e.g., Abbott & Volberg, 1999; Blaszczynski & McConaghy, 1994a; Laursen et al., 2016). The non-violent nature of crimes related to gambling is also in general confirmed by the review conducted by Adolphe et al. (2018), although according to the authors, violent crimes may also occur at a higher-than-expected rate.

Methods of measuring the prevalence of criminal activity associated with pathological gambling are varied, often problematic, and differ from study to study (making it difficult to compare the results of studies with each other). A relatively frequently used method is surveys of prisoners and offenders, e.g., study on 332 male inmates in Japan done by Yokotani et al. (2019), similar research done by Pastwa-Wojciechowska (2011) in Poland, or Abbott et al. (2005) in New Zealand. Other studies focus on pathological gamblers who have opted for treatment – for example, the study done by Meyer and Stadler (2009), where 89.3% of people seeking treatment confessed to gambling-related crime or study of Blaszczynski and McConaghy (1994a, 1994b) who reported 59% of such respondents. Among those calling Argentinian specialized helplines, 32% reported engagement in illegal activities (Folino & Abait, 2009). These studies are problematic, obviously because of the selection bias and the small sample, however, research conducted on the general population is limited by underreporting, because crimes are often committed against family members who do not report them to authorities (as Sakurai & Smith, 2003 and Adolphe et al., 2018 point out). Another problem that makes it difficult to compare prevalence findings between studies is that different authors use different definitions of problem gambling (by frequency of gambling, by impaired life functioning, or by level of preoccupation), so the results vary (see Blaszczynski & Nower, 2002; Vitaro, et al., 1999).

One of the general motives in terms of motivation to engage in criminal activities is chasing losses (see Lesieur, 1984), regardless of the player's financial security (Sakurai & Smith, 2003). Players often raise the stakes when trying to recover, getting themselves under stress in the process. Subsequently, when the original sources of financing are exhausted, criminal acts seem to be the only way to escape the resulting crisis (Blaszczynski et al., 1989; Turner et al., 2009). Another source of motivation to commit crime due to gambling is (according to Blaszczynski & McConaghy, 1994b) the need to meet one's financial obligations. In Spunt’s (2002) study, two-thirds of respondents said they had committed crimes to pay off gambling debts and one-fourth to generate more money to feed their gambling habits. Another explanation is Agnew’s (1992, 2001) general strain theory, which describes that the individual comes under various pressures (not only financial, but also family, social, etc.) and criminal activity represents a means of coping. Thus, if gambling brings these strains, the individual may be motivated to commit crime as a side effect (Abbott & McKenna, 2005; Adolphe et al., 2019; Turner et al., 2017).

Various studies described factors that are correlated both with problem gambling and tendencies to commit crime. Typically, these are male young adults, socially disadvantaged, often from ethnic or racial minorities (Welte et al., 2011, 2017; DeLisi & Vaughn, 2016; Bergen et al., 2012), including individuals with a history of drug abuse (Barnes et al., 2015; D’Amico et al., 2008; Ladouceur et al., 1999). Furthermore, a common factor is poor academic performance and employment during adolescence (Canale et al., 2016; Ladouceur et al., 1999; Uggen & Wakefield, 2008), low attachment to one’s own parents and school, being close to delinquent peers (Dowling et al., 2017; Hoeve et al., 2012; Welte et al., 2017), and low self-control and depression (Bergen et al., 2012; Welte et al., 2017; DeLisi & Vaughn, 2016).

Given the task of this paper, we can find studies evaluating the effects of supply reduction on the prevalence of gambling in a given area (see review by Meyer et al., 2018). Delfabbro (2008) studied the results of South Australia’s decision to remove 2,168 gaming machines (proportionally to the size of the venue). Out of 400 respondents, 7.5% answered that they altered their gambling behavior, 80% of them played less, 43.3% spent less money on gambling and 46.7% spent less time on gambling activities. In the USA Carr et al. (1996) found, that during the 3-month long ban of gambling machines in South Dakota, the number of inquiries from and treatments of gamblers rapidly fell. After the ban was lifted, the numbers rose again (although to lower level than before). Welte et al. (2016) studied the impact of reduction of permitted gambling options in different US states. Their results indicated that the proportion of problem gamblers fell by 7.5 to 14.6 percentage points (depending on the number of gambling options reduced). Norway introduced nationwide ban on electronic gambling machines for a limited period of time in 2007, which according to findings of Lund (2009) resulted in significant fall of prevalence of almost all forms of gambling, with the exception of internet gambling. Bu and Skutle (2013) reached a similar result in their research among gamblers seeking help in treatment centers – a decline in playing slot machines, but an increase in sports-betting and internet-based gambling.

Unfortunately, many of these studies suffer from small (and selective) samples, limited applicability of results, and most importantly, they usually do not use control group, control variables or some sort of econometric technique or identification strategy to confirm causality (see Meyer et al., 2018). One of the exceptions is for example the study of Kato and Goto (2017), who evaluate the relationship between geographical accessibility and prevalence to pathological gambling in Japan using the instrumental variable approach. Other exception is the study by Bottan et al. (2017). They examined crime in Illinois, USA, where gambling is permitted, but local municipalities were allowed to ban gambling within their jurisdiction (which the City of Chicago did in case of video gambling). Difference-in-differences strategy exploited this set-up by comparing crime in areas closer and further away from gambling establishment (before and after the ban) and found that access to gambling indeed increased property and violent crimes persistently over time.

This study will work with a similar set-up but given the limitations of the difference-in-difference and instrumental variable approach, will use the synthetic control method. This is a relatively new and popular method introduced by Abadie and Gardeazabal (2003) which was successfully used, for example, to analyze German reunification after the fall of the Iron Curtain (see Abadie et al., 2015), the impact of Brexit on UK’s financial markets (see Opatrny, 2021), or the impact of economic liberalization (see Billmeier & Nannicini, 2013). This data-driven approach brings with it many advantages, most notably a high-quality counterfactual that allows for a robust assessment of the impact of a given policy (see the methodology section of this paper). This method has been called *"arguably the most important innovation in the policy evaluation literature in the last 15 years*." by Athey and Imbens (2017, p. 9). As far as we are aware, this is the first time this method is used in the context of gambling, and our study serves as a proposal to include this method in the toolbox of methods used to investigate the impact of a given policy.

## Background

Gambling and its related industry experienced a great development in the Czech Republic after the fall of the communist regime in 1989. However, it is only in recent years that a scientific and public debate has emerged regarding the far-reaching effects of gambling on an individual and social level (see Winkler et al., 2017 and Szczyrba et al., 2015). In 2017, an amendment to the Lottery Act (Act No. 186/2016 Coll., on Gambling) came into effect, which affected online gambling, the legal circumstances of the Czech Republic’s accession to the European Union and other aspects. According to the latest survey (Mravčík et al., 2021), approximately 35–50% of adults in the Czech Republic reported they had gambled at least once in the past twelve months. The most common types of gambling are lotteries, land-based sports betting (5–15% in the last twelve months), online sports betting (3–17%) and land-based technical games (3–6%). Problem gamblers account for 2.4% (according to Lie/bet scale) and 4.5% of the population (PGSI scale). For those at high risk, both scales produce comparable prevalence results, i.e., about 1.3% of the population. (Mravčík et al., 2021) Across the studies, it appears that the highest number of problem gamblers is among users of land-based technical games and fixed-odds betting (including online). (Mravčík et al., 2021) All data on problem gambling in the Czech Republic are consistent with the DSM-5 definition (formerly referred to as pathological gambling in the DSM-IV definition). Last but not least, there is one other factor, that could be relevant regarding gambling, Czech Republic is one of the most secular and least religious countries not only in Europe, but in the whole world (Pew Research Center, 2017). Thus, Czech gamblers experience little or no religious stigma associated with gambling (which is felt, for example, by Muslim or Protestant believers, see Uecker and Stokes (2016)).

Regarding gambling-related crime, according to the 2019 Survey of Gamblers in Treatment (unpublished study, see Mravčík et al., 2021), 51% of pathological gamblers have ever committed theft (43% in 2017), 37% of Czech pathological gamblers have committed fraud (29% in 2017), 33% have committed embezzlement (27% in 2017), 28% have engaged in manufacturing and/or selling illicit drugs (24% in 2017), and 12% have committed robbery (8% in 2017). The link between gambling and secondary crime in the Czech Republic can also be drawn from a questionnaire study conducted among incarcerated individuals (unpublished study, see Mravčík et al., 2021) performed in 2020, where 20.4% of respondents in prison stated that they had committed theft as a result of gambling or to pay off debts as a result of gambling.

The aforementioned Lottery Act of 2017, among other measures, allowed municipal representatives to restrict or completely ban land-based gambling in their territory through the instrument of generally binding regulation (GBR). Czech municipalities are using this tool to reduce the supply of gambling in their territory given the aforementioned trends linking gambling and crime, and due to the generally negative view of the general public on the issue of gaming parlors and casinos. At the moment, a total of 709 municipalities in the Czech Republic regulate gambling through the GBR, of which 444 municipalities have completely banned technical games (slot machines, IVTs, etc.). This includes 35 of the most populous cities and 59 out of 61 municipalities over 20,000 inhabitants (MFCR, 2022).

This environment creates ideal conditions for using the synthetic control method to examine the impact of a local gambling ban on crime. Although research has been done regarding the prevalence of gambling and other addictology aspects, general description of specific factors of Czech environment (e. g. Szczyrba et al., 2015), social costs of gambling (Winkler et al., 2017) or regional aspects of gambling (Fiedor, 2017), there is a lack of sophisticated analyses of association of gambling with crime or impact studies in general. At the same time, in virtually no municipality that has used GBR, an ex-ante analysis of the impact of the ban has been conducted. Thus, not only will this study be the first to use the synthetic control method in this context, it will also be the first study to examine this issue in the Czech setting.

## Methodology

The main principle of the synthetic control method is to construct a synthetic counterfactual that closely matches the treated group before the intervention (treatment event). The panel data should consist of at least one treatment unit and a number of untreated control units (sometimes called "donor pool"), the resulting synthetic control group is then a weighted average of these units from the donor pool. If a close match between the control and treatment groups in the pre-treatment period is established and there is a significant difference between the observed variable between the two groups after the intervention, there is an impact. (Abadie & Gardeazabal, 2003).

This method has several advantages over the difference-in-differences approach used in impact studies with a similar setting. The main difference-in-differences assumption (which is often quite challenging) is the common trend assumption, which in the case of the synthetic control method is replaced by forming a synthetic control group that fits the pre-treatment period as tightly as possible (see below). Furthermore, the synthetic control method requires only one treatment unit to be used and also accounts for the effects of confounding factors varying over time (Kreif et al., 2016).

Suppose a sample of *J* + *1* units. Only the first unit faces the intervention of the treatment (a given policy), the remaining *J* units serve as control units (the previously mentioned donor pool). As Abadie et al. (2015) mention, it is important to ensure that those remaining units are thought to be driven by the same structural process as the treated unit. For example, Abadie and his colleagues used West Germany as a treatment unit and other OECD countries as controls. Analogically, our study will use a Czech district city with a land-based gambling ban and the control group will consist of other Czech district cities without the ban (see details below). The sample is a balanced panel with all units observed at the same time periods *t* = *1, …, T*. The outcome variable is observed in both pre-intervention (${T}_{0}$) and post-intervention (${T}_{1}$) periods ($T={T}_{0}+ {T}_{1}$). Logically, we assume that the intervention has no impact on both groups during the pre-treatment periods, as the impact should occur during periods ${T}_{0}$ + *1, …, T* and only to the treated unit. Therefore, the treatment effect is the impact of the intervention on the observed variable during the post-intervention periods. If we denote potential outcome without the intervention in the unit *i* in period *t* as ${Y}_{it}^{N}$ and ${Y}_{it}^{I}$ as the outcome affected by the intervention in the unit *i* in period *t*, the effect of the treatment intervention in post-intervention period (as in pre-intervention period those two outcomes should be the same) is represented by:

$${v}_{it}={Y}_{it}^{I}-{Y}_{it}^{N}$$

The main task is to find the weighted average of the units in the donor pool to construct the synthetic control group. Abadie and Gardeazabal (2003) in their original paper defined such weights as $w={\{w}_{2}, \dots , {w}_{J+1}\}$ and

$$\sum_{j=2}^{J+1}{w}_{j}=1$$

where *j* = *2, …, J* + *1*. Therefore, the estimators of ${v}_{it}$ and ${Y}_{it}^{N}$ can thus be written as

$${\widehat{v}}_{it}={Y}_{it}^{I}-{\widehat{Y}}_{it}^{N}$$

$${\widehat{Y}}_{it}^{N}={w}_{2} {Y}_{2t}+\dots + {w}_{J+2}{ Y}_{J+1, t}$$

Regarding the weights of the control units themselves, Abadie et al. (2010) choose such weights that minimize

$${v}_{1}{\left({X}_{11}-{w}_{2}{X}_{12}-\dots -{w}_{J+2}{X}_{1,J+1}\right)}^{2}+\dots +{v}_{k}{\left({X}_{k1}-{w}_{2}{X}_{k2}-\dots -{w}_{J+2}{X}_{k,J+1}\right)}^{2}$$

where the key element of the calculation is to find relative importance of the synthetic control assigned to predictors: ${\{v}_{1}, \dots , {v}_{k}\}$. As in many such studies (e.g., Opatrny, 2021), our choice is to find such weights that minimize the prediction error (the cross-validation technique). This could be done by minimizing the root mean square predicted error (RMSPE):

$${\text{RMSPE}}={\left(\frac{1}{{T}_{0}}\sum_{t=1}^{{T}_{0}}{\left({Y}_{1t}-\sum_{j=2}^{J+1}{w}_{j}*{Y}_{jt}\right)}^{2}\right)}^\frac{1}{2}$$

It is worth noting that in practice synthetic control can be calculated using freely available scripts for either R, Stata or MATLAB that generate these weights (see Abadie et al., 2015).

Although this method is quite versatile, several conditions need to be met for its successful use. Along with the use of the balanced panel mentioned above, unit with similar treatment should not be included in the data (to avoid bias in the output). Second, control units should be somewhat similar in performance with the treatment unit to achieve a good fit. Moreover, it should be taken into account that some control units could be affected by the intervention (for example by proximity to the treated unit), so those should also not be included in the dataset to avoid interpolation bias. And finally, it is recommended to not use the synthetic control method if the control and treatment group in the pre-intervention period do not fit properly or when the number of pre-treatment periods is too small. (Abadie et al., 2015).

Regarding statistical inference, the situation is rather problematic. Abadie et al. (2015) mention that because of relatively small sample, the absence of randomization and given the fact that sample units are not selected through probabilistic sampling, standard approaches could not be fully used. Nevertheless, there are several widely used methods to test counterfactuals in the synthetic control method – mainly the so-called "placebo tests" (first introduced by Abadie & Gardeazabal, 2003). These are generally based on the intuition that if the effect occurs even for units that are not affected by the intervention (or if the effect is even greater), this will contradict the assumption about the effect of the intervention altogether. In other words, if in our case the effect of the gambling ban is shown to be equal or greater in cities where it was not actually banned, it will greatly diminish the confidence of the effect of the gambling ban itself.

Therefore, the effect of the intervention in the treated unit (${\widehat{v}}_{1t}$) is compared with the effect of the intervention in the control unit (${\widehat{v}}_{it}$) for each unit *i* and period *t*. Abadie et al. (2015) recommend to use following statistic and its distribution:

$${{\text{RMSPE}}}_{i}=\frac{\sum_{t={T}_{0}+1}^{T}{\left({Y}_{it}-{\widehat{Y}}_{it}^{N}\right)}^{2}/\left(T-{T}_{0}\right)}{\sum_{t=1}^{{T}_{0}}{\left({Y}_{it}-{\widehat{Y}}_{it}^{N}\right)}^{2}/{T}_{0}}$$

Also, an empirical p-value could be constructed:

$$p= \frac{b+1}{N+1}$$

where *b* represents the number of placebo estimates, for which the effect is equal to or greater than the effect estimated for the treated unit, and *N* represents the total number of placebo tests. Simply put, it is the proportion of units (placebo and treated) that have the same or greater effect than the treated unit. If the p-value is equal to zero, it is absolutely certain that the effect for the treated unit (the city with the gambling ban) is significantly higher than the effect for control units (cities without the ban). Thus, this p-value can also be compared to some predefined level of significance to reject the null hypothesis of no effect of the intervention. As a robustness test, Abadie et al. (2015) also recommend sequentially removing significant control units and re-estimating the model to see if the model is driven by any particular control unit. The robustness could be also checked by removing the control variables in the similar manner. For other inference methods regarding synthetic control method, see Firpo and Possebom (2018).

## Data

Building on the Background chapter, we will exploit the set-up where land-based gambling is banned only in some cities in the Czech Republic. The sample is restricted to the main cities in their districts (Czech districts are statistical regions on the LAU 1 level) that have banned gambling through the GBR. In addition, we selected the treated cities based on the fact that they have reduced the number of land-based games to zero in roughly the same time period (the beginning of 2017). In total, we used four treated district cities – Klatovy, Domažlice, Písek and Český Krumlov. All of them have a comparable population (between 11,000 and 31,000 inhabitants). As control cities, we used Czech district cities that do not have GBRs in place at all or have cancelled them, and that had land-based games in operation for the entire period 2013–2021, so the ban does not apply to them. We selected a total of eleven such cities (Trutnov, Hodonín, Opava, Prachatice, Tábor, Jeseník, Nymburk, Strakonice, Příbram, Semily and Vyškov). Since we have four treated cities, we will thus present the results of four separate models through the synthetic control method.

As the outcome variable, we used the crime index developed by Otevřená společnost, o.p.s. initiative. The data is available through an interactive web map (see Otevřená společnost, 2022) that contains comprehensive database of all crimes committed at the level of the individual police department (i.e., the smallest independent unit). The resulting crime index represents the number of crimes per 10,000 inhabitants in a given area. The data from this map is available on a monthly basis since 2013, so a time series of the crime index for each monitored area at the city level can be obtained very efficiently. Although the data on types of crimes (theft, robbery, drug production, etc.) can be attained, we used the overall crime index because the data for these crimes are unfortunately incomplete, and it would not be possible to obtain a balanced panel. However, within the control cities there is a correlation between this index and the number of land-based technical games, so the overall crime index serves as a usable proxy for the crime rate partially caused by gamblers.

We used unemployment, average wage, education and population density as control variables explaining the crime rate (we constructed a base index (2013 = 100) for unemployment, average wage and population density). Not all of these variables are available at the city level, unemployment data are only available at the district level (LAU 1 level) and average wage and education at the level of region (NUTS 3 level). All of these data were obtained from the Czech Statistical Office (CZSO, 2022).

The resulting panel thus consists of fifteen cities (four treated cities and eleven control cities forming the donor pool) between 2013 and mid-2021. All variables are available at half-yearly intervals, so we have seventeen observations for each unit. Each time, the series is divided into a pre-treatment period (eight observations until the beginning of 2017) and a post-treatment period (nine observations). See Table 1 for basic descriptive statistics of mentioned variables.

**Table 1:** Descriptive statistics of variables

	Mean	Standard deviation	Min	Lower quartile	Median	Upper quartile	Max
Crime index	17.174	6.644	2.818	12.690	16.103	21.077	50.683
District unemployment (2013 = 100)	0.051	0.024	0.013	0.031	0.047	0.068	0.119
Region average wage (2013 = 100)	1.232	0.171	1.000	1.065	1.182	1.393	1.556
Low education ratio	0.211	0.081	0.073	0.164	0.197	0.242	0.565
Population density (2013 = 100)	0.989	0.016	0.924	0.980	0.991	1.000	1.022

*Source*: own calculation

## Results

As explained above, out dataset contains a total of four Czech district cities with a ban on land-based gambling, so we will present four models estimated using synthetic control method. For all four treated cities, we first use this method to construct a synthetic counterfactual (built from the donor pool of eleven cities mentioned above) that represents what the crime index would have looked like in the absence of the ban on land-based games (the intervention). If there is a tight fit in the pre-intervention period, we will compare the outcomes in the post-intervention period and assess whether or not there was a significant impact. Afterwards, we will test robustness of these models, and we will also run placebo tests described by Abadie et al. (2015). All results presented in this paper were obtained in R using the free script mentioned above (using *Synth* and *SCtools* packages) or in the freeware software Gretl.

**Fig. 1:**
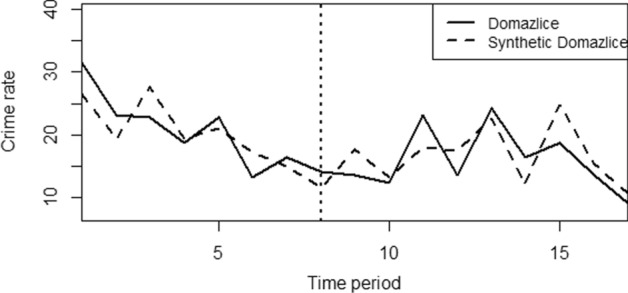
Trends in crime rates in Domažlice and in synthetic Domažlice obtained by the synthetic control method (half-yearly data, first period = first half of 2013). *Source*: own calculation

**Fig. 2:**
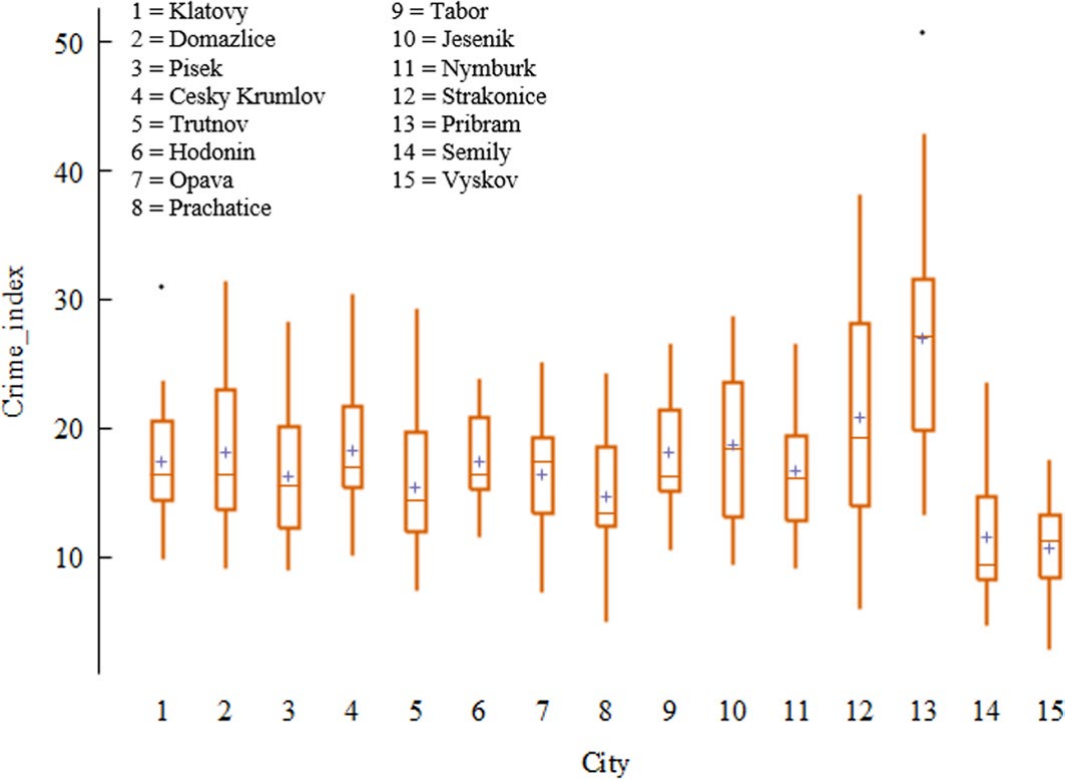
Distribution of crime index by city. *Source*: own calculation

We start with the city of Domažlice. As we can see in Fig. 1, thanks to the synthetic control method we achieved fairly tight fit in the pre-treatment period (dotted vertical line indicates intervention—the moment when the city reached zero technical games at the start of 2017):

A similarly tight fit is achieved in the post-treatment period, so at first glance the ban on land-based gambling in Domažlice had no effect on crime, as the crime rate would have had a similar trend in the hypothetical case without the ban. As can be seen in Table 2, this result is mainly driven by the city of Nymburk (weight 50.2%). Therefore, as a robustness test, we first removed Nymburk from the original model and subsequently removed Prachatice (with the highest weight of all cities (41.5%) in the reduced model) from this reduced model. Next, we removed the population density variable from the original model, whose weight as a control variable is 84.6% (see Table 2), and finally, we removed the cities of Strakonice, Příbram, Semily and Vyškov, which showed too much heterogeneity in the crime index compared to other cities (see Fig. 2).

**Table 2:** Weights of individual cities and control variables for the Domažlice model calculated by synthetic control method

City	SCM weight	Variable	SCM weight
Trutnov	0.065	Unemployment rate	0.000
Hodonín	0.006	Average wage	0.154
Opava	0.005	Low education	0.000
Prachatice	0.007	Population density	0.846
Tabor	0.008		
Jesenik	0.006		
Nymburk	0.502		
Strakonice	0.385		
Pribram	0.005		
Semily	0.005		
Vyskov	0.005		

*Source*: own calculation

**Fig. 3:**
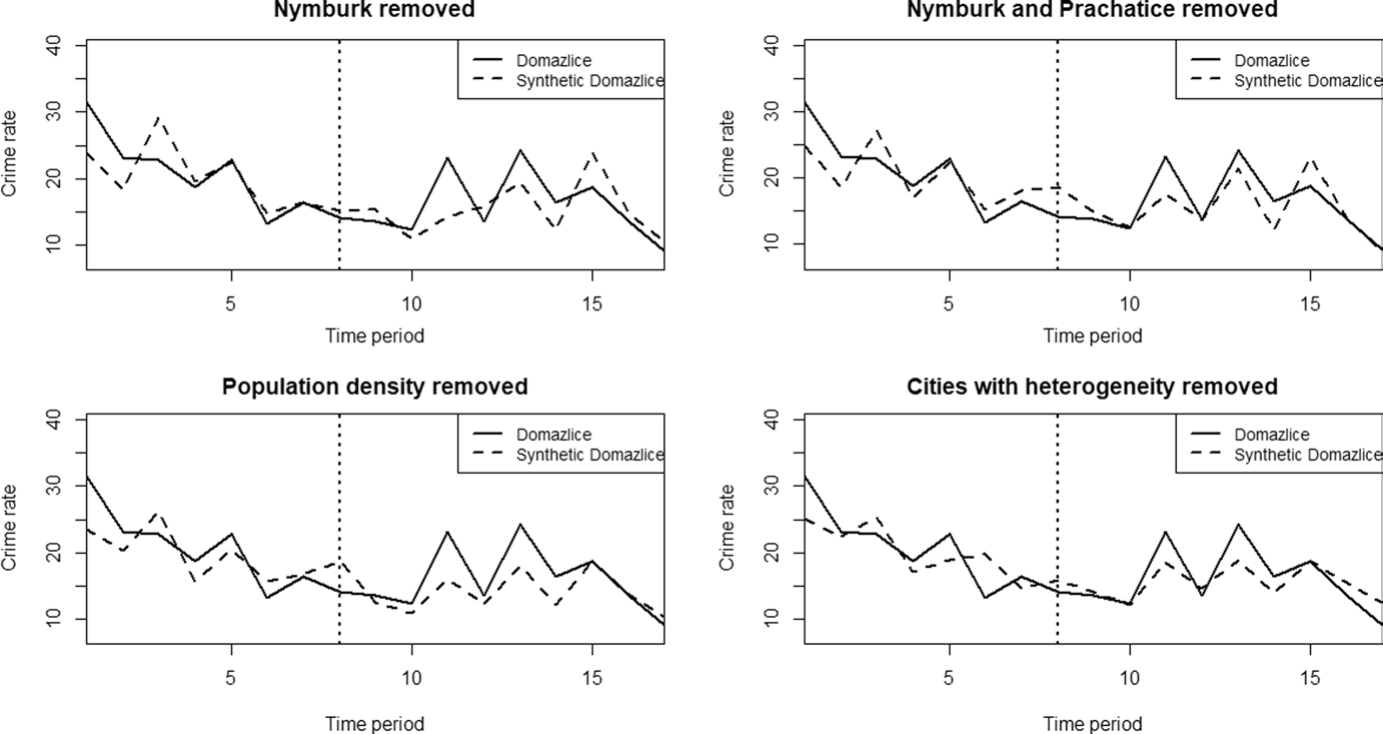
Robustness tests of the Domažlice model. *Source*: own calculation

Figure 3 summarizes the path of the synthetic and real crime index when the mentioned cities and variables are removed. As we can see, the synthetic outcome and their tight fit with real outcome in all graphs is more or less identical, so the model proved to be robust as the results were not overly driven by any single city or variable.

**Fig. 4:**
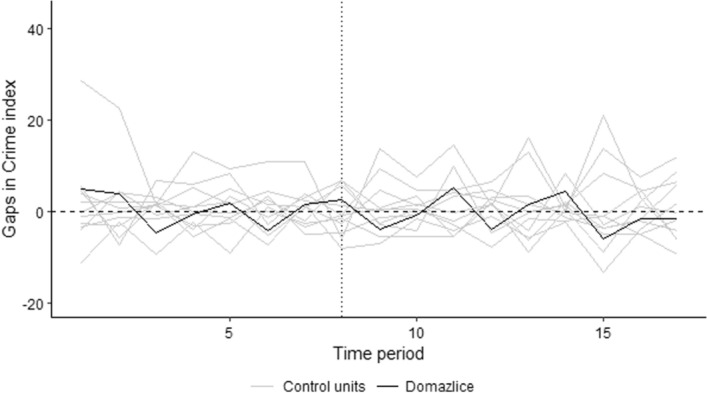
Placebo tests of the Domažlice model. *Source*: own calculation

Next, we ran placebo tests. As described above, we simulated the effect of the gambling ban on all control cities. As Fig. 4 shows, the intervention effect is larger in many other control cities than in the case of treated Domažlice (dotted vertical line again indicates intervention).

The resulting empirical p-value of 0.667 implies that even at the 10% significance level, the null hypothesis of no effect of the gambling ban on crime in the city cannot be rejected. Therefore, every approach we tried (both robustness and placebo tests) shows the same results – that the ban on land-based in Domažlice had no effect on crime in this city.

**Fig. 5:**
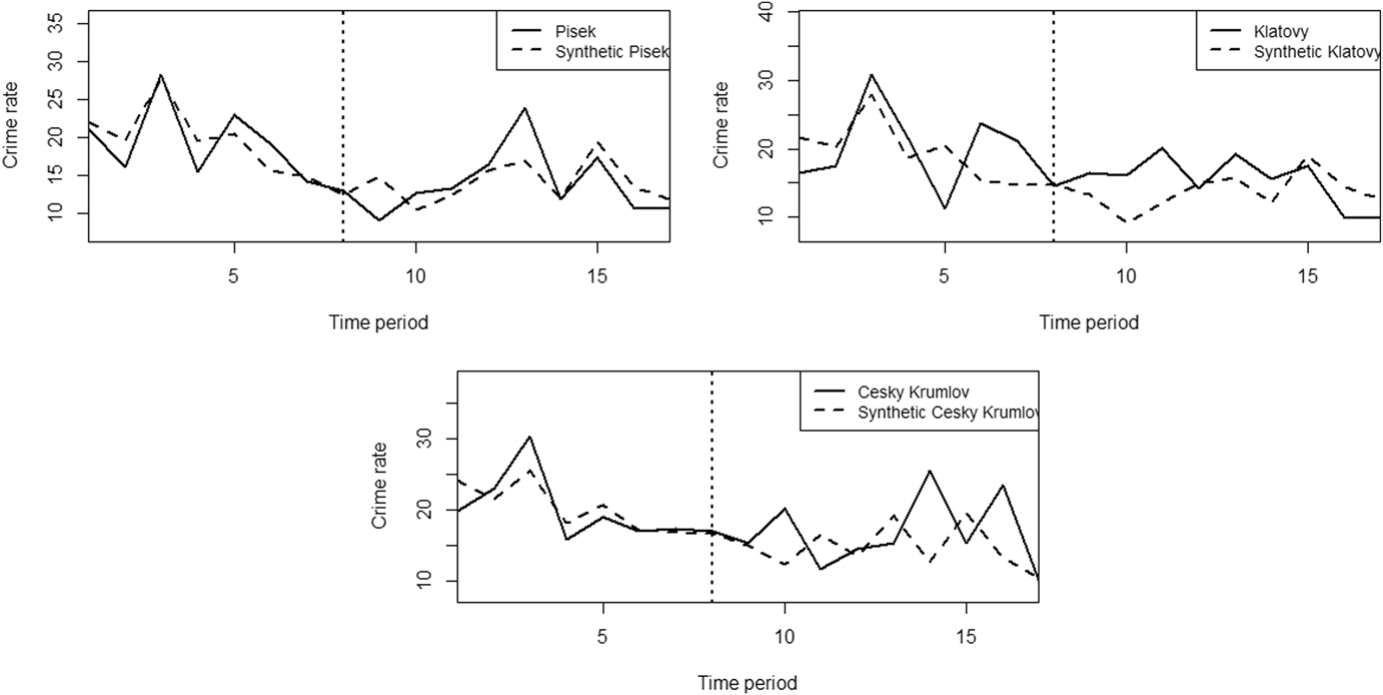
Trends in crime rates in Písek, Klatovy and Český Krumlov and in synthetic Písek, Klatovy and Český Krumlov obtained by the synthetic control method (half-yearly data, first period = first half of 2013). *Source*: own calculation

Since the procedure is the same for the remaining three cities tested, to save space we will present the results for the remaining three cities together. We started again with applying synthetic control method to the models with all eleven control cities and four control variables. Figure 5 shows that the synthetic control method generated a good pre-treatment counterfactual for both Písek, Klatovy and Český Krumlov. In all three cases, there are short post-treatment periods in which the synthetic outcome appears to deviate somewhat downward from the real outcome.

**Fig. 6:**
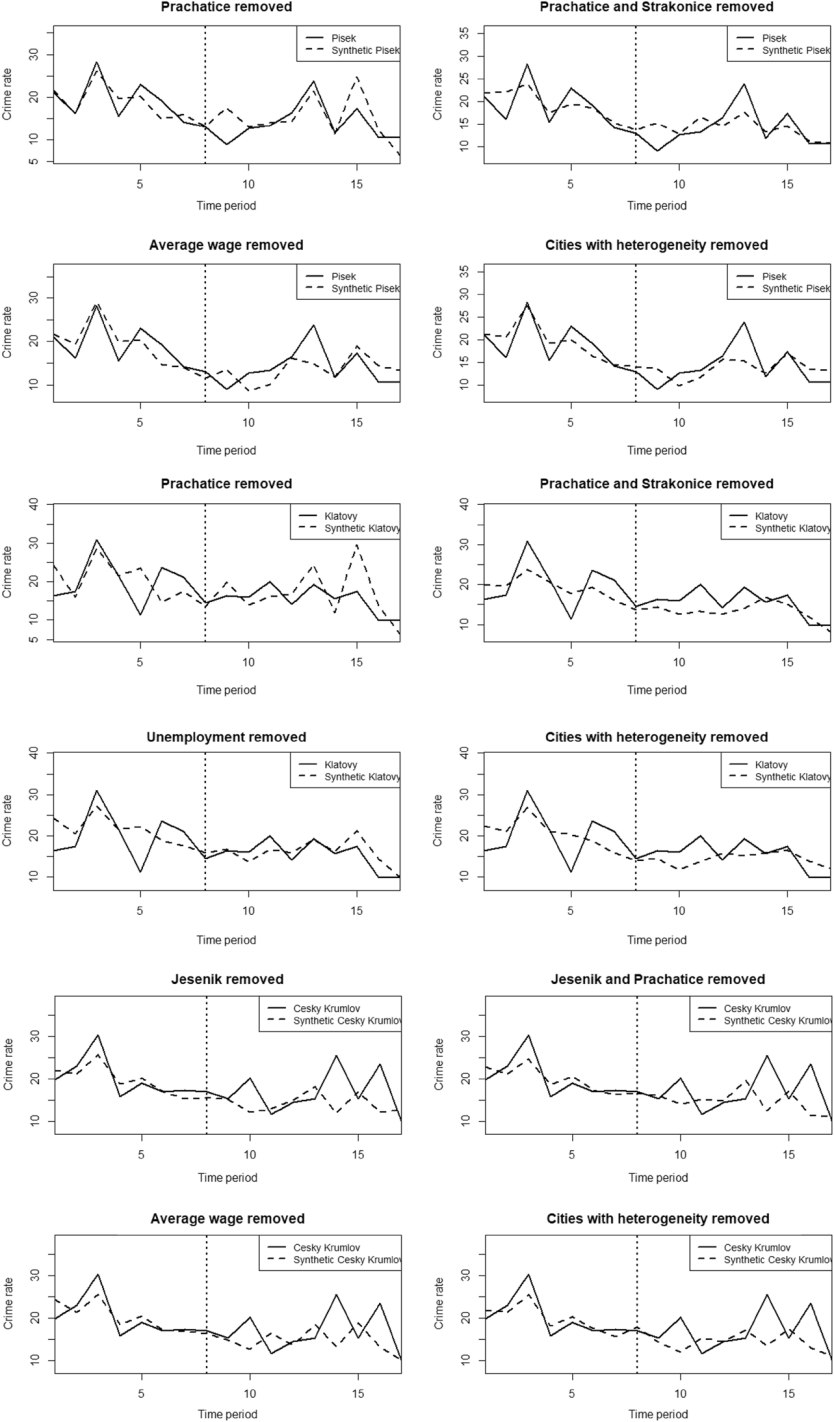
Robustness tests of the models of Písek, Klatovy and Český Krumlov. *Source*: own calculation

We again apply robustness and placebo tests to examine the models and test the hypotheses about the effect of the intervention on the outcome variable. As in the case of Domažlice, we removed (1) the control city with the largest weight (see Table 3) in the synthetic counterfactual of the original model, (2) the control city with the largest weight in the subsequent reduced model, (3) the control variable with the largest weight in the original model, and (4) the aforementioned four cities due to high heterogeneity in the crime index. According to the resulting twelve graphs (see Fig. 6) we can again confirm the robustness of the models, as the outcomes have not changed significantly from the original state.

**Table 3:** Weights of individual cities and control variables for the Klatovy, Písek and Český Krumlov model calculated by the synthetic control method

City	SCM weight	Variable	SCM weight
*Klatovy *			
Trutnov	0.090	Unemployment rate	0.264
Hodonín	0.007	Average wage	0.689
Opava	0.030	Low education	0.008
Prachatice	0.515	Population density	0.038
Tabor	0.062		
Jesenik	0.001		
Nymburk	0.046		
Strakonice	0.142		
Pribram	0.019		
Semily	0.029		
Vyskov	0.059		
*Písek*			
Trutnov	0.030	Unemployment rate	0.658
Hodonín	0.001	Average wage	0.337
Opava	0.012	Low education	0.000
Prachatice	0.648	Population density	0.005
Tabor	0.030		
Jesenik	0.174		
Nymburk	0.014		
Strakonice	0.042		
Pribram	0.009		
Semily	0.014		
Vyskov	0.027		
*Český Krumlov*			
Trutnov	0.076	Unemployment rate	0.000
Hodonín	0.061	Average wage	0.610
Opava	0.091	Low education	0.353
Prachatice	0.089	Population density	0.037
Tabor	0.089		
Jesenik	0.207		
Nymburk	0.068		
Strakonice	0.090		
Pribram	0.069		
Semily	0.100		
Vyskov	0.061		

*Source*: own calculation

**Fig. 7:**
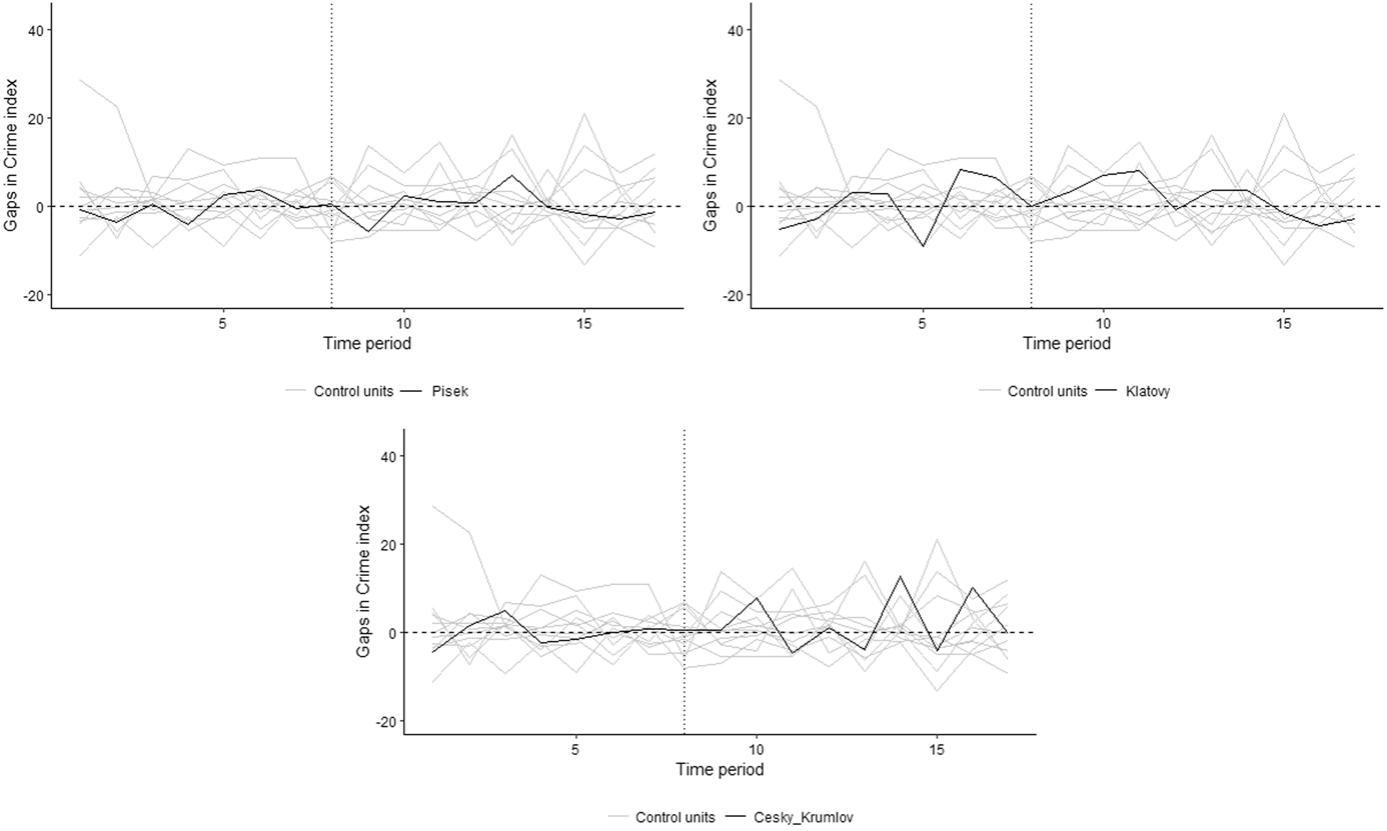
Placebo tests of the Písek, Klatovy and Český Krumlov model. *Source*: own calculation

And finally for all three cities we run placebo tests, see Fig. 7 for the results:

Although all cities seemed to show some effect of the ban on land-based gambling, all graphs indicate that the intervention effect is again larger for many other control cities. This is confirmed by the calculated empirical p-values: 0,333 for Písek, 0,833 for Klatovy and 0,167 for Český Krumlov. Therefore, even for these cities, we cannot reject the hypothesis of no effect of the intervention (banning land-based gambling) on crime.

In conclusion, using the synthetic control method, we did not find a significant effect of the ban on crime in any of the cities examined where the ban was implemented through the GBR. All treated cities in our sample have approximately the same crime rate they would have even without the ban.

## Discussion and limitations

The specific conditions of the Czech setting allowed us to take advantage of the strengths of the synthetic control method. Our data meet the conditions outlined by Abadie et al. (2015) and Abadie and Gardeazabal (2003). The donor pool is driven by the same structural process as the treated units, as all cities come from the same country, which provides the same institutional environment, economic shocks, etc. At the same time, we used cities with the same ban period (the same period of reduction of technical games to zero), which makes this intervention more comparable across treated cities. We also ensured that control cities had a stable supply of land-based gambling throughout the observation period to meet the assumption that no control unit (city) was affected by the intervention. Last but not least, we used a total of four treated cities to avoid selection bias and to avoid having a possible effect (or no-effect) tied to only one location.

But despite all these efforts, and despite all the robustness and placebo tests, selection bias cannot be completely avoided because our data consist of cities that were selected on the basis of the enactment of the GBR. There are also some potentially influential individual characteristics of certain cities – for example, Český Krumlov is a UNESCO World Heritage Site. Therefore, we introduced variables controlling for city size, economic situation and education into the model (in accordance with for example Bergen et al., 2017 or Canale et al., 2016). Another potential limitation is overly broad definition of the crime index (see above) or the availability of some variables used in the model only at the level of higher administrative units. Finally, a longer pre-treatment period would have improved the estimated model; in this respect, we were limited primarily by the crime index, which began to be collected in 2013, yet this is still a sufficiently long time period. To summarize, our main limitations regarding the use of the synthetic control method are the choice of treated and control units and the available data.

The main objective of this paper is to introduce the synthetic control method as a suitable analytical tool to study gambling and its impacts. This method allows us to avoid the fundamental problems associated with finding causality (like e.g., in case of prisoners’ surveys done by Yokotani et al., 2019) described by Meyer et al. (2018) and use an identification strategy to actually find the impact of a given measure. At the same time, the synthetic control method is in many cases more useful than the instrumental variable approach (used by Kato & Goto, 2017) or the difference-in-differences approach (see Bottan et al., 2017), as these two approaches are even more dependent on the specific situation and assumptions. As our study shows, the synthetic control method is a relatively versatile and easy-to-use method that yields well-presentable results and although our study is based on the specificity of the Czech environment, as Firpo and Possebom (2018) show, it is applicable to a wide range of impact studies and situations.

Regarding the second main objective of this paper, i.e., testing the effect of a gambling ban on crime in the Czech Republic, although it is argued that problem gambling is related to crime (in line with Adolphe et al., 2018; Banks, 2017, and others), as the case of Czech cities shows, banning this activity may not lead to a decrease in crime. There are many close substitutes to land-based games that have emerged especially in the era of internet and mobile gaming. In the Czech environment, Mravčík et al. (2021) and other researchers indeed talk about a shift of players to the Internet; other substitutes to which pathological gamblers may resort include the black market for gambling (both land-based and online, see Vobořil, 2020). Another partial explanation may lie in the commuting of gamblers outside their municipality, where gambling is banned through the GBR, to municipalities where it is not. Yet another useful observation is the trend in the prevalence of gambling in the Czech Republic during the Covid-19 pandemic when land-based gambling facilities were closed for a large part of 2020 and 2021 – this period de facto simulated a total prohibition of land-based gambling. As Mravčík et al. (2021) points out, while the general prevalence of gambling has declined year-on-year between 2019 and 2020, some data show that the prevalence of pathological gambling has increased slightly. This may imply that in an environment without land-based gaming, pathological gamblers have found a substitute, whether in the form of online gaming or other activities, but comprehensive research is still needed in this regard. In any case, this conclusion (although not yet confirmed) would contradict the findings of Welte et al. (2016) or Lund (2009).

So the question remains as to why this study is one of the very few to refute a causal relationship between gambling prohibition and a decline in crime. One obvious explanation may be the substitution of prohibited land-based gambling by its online variant (and by above-mentioned commuting to other areas etc.), enhanced by the widespread use of smartphones. Crime may simply not have fallen because gamblers moved from gambling halls and casinos to the internet (compared to other studies conducted before the proliferation of online gambling). However, the ban on gambling in the observed municipalities took place in 2017, when online gambling took up just under a quarter of the Czech market (see Mravčík et al. (2021)), so online gambling was not as significantly prevalent at that time. However, the gradual increase in land-based gambling bans in municipalities may have contributed to the gradual increase in the share of online gambling in the overall market, but we do not have the data to answer this hypothesis. Another explanation may be the different definitions of problem gambling (DSM-5 and CPGI definitions), or different metrics for measuring the prevalence of problem gambling (Lie/bet scale and PGSI), or in the definitions of individual crimes. In our study, we did not use self-reported crime (as e.g. Delfabbro (2008) did), nor did we use interviews of gamblers in treatment, but we used similar data to Bottan et al. (2017), i.e. police data on individual crimes. Thus, a possible explanation for the different result may be differences between US and Czech criminal law, or possibly a difference in the proportion of underreported crimes (but since both are crimes related to gambling, underreporting should be similar). Yet another part of the picture could be explained by the above-mentioned high level of secularization of Czech society and other possible unobserved characteristics of Czech gamblers. Therefore, the answer to why causality is unproven probably lies in several factors mentioned above—the increasing possibility of substitution of land-based gambling in recent years, possible specific conditions of the tested territory and society, different definitions and others. However, the main difference between our study and those of other authors is the use of the synthetic control method.

## Conclusion

We used the synthetic control method developed by Abadie and Gardeazabal (2003) to examine the effect of land-based gambling ban on crime. We exploited a set-up where Czech municipalities have the authority to ban land-based gambling on their territory. Using panel data on a sample of four cities that have implemented the ban and eleven control cities, we show that the ban had no effect on crime in these cities (controlling for wages, education, and population density). In other words, even though crime may be related to gambling, the crime rate development in examined cities would be roughly the same if there were no gambling ban in the city. This was confirmed by robustness tests as well as placebo tests developed by Abadie et al. (2015). The synthetic control method allowed us to easily construct a high-quality counterfactual and, when used correctly with an appropriate inference method, it can be used as an effective identification strategy when investigating causality.

This study is the first to use this relatively new and well-developed analytical tool to examine gambling and its impact. Therefore, its contribution lies not only in exploring the impact of this policy on crime (moreover, for the first time in the Czech Republic), but more importantly in demonstrating the possibilities of using this method in this context. Many similar studies suffer from small sample sizes, the absence of a control group or control variables, and an overall lack of applicability of the results. We believe that more frequent use of this and similar methods (e.g., the difference-in-differences approach) can improve this situation. Further analyses can thus address both the use of the synthetic control method to model other gambling-related issues and the impact of gambling bans on crime in other countries (e.g., due to closed land-based establishments because of Covid-19). Moreover, our study is one of the few to refute causality between gambling prohibition and crime reduction. Unfortunately, we do not have the data to show why this is the case—it could be due to greater substitution possibilities of the banned land-based gambling, specific conditions of a given territory (i.e. the Czech cities studied), or different definitions in terms of pathological gambling or crime. This opens up further possibilities for future research.

## Data Availability

The data that support the findings of this study are available from the corresponding author upon reasonable request.
